# Immersive Virtual Reality and Virtual Embodiment for Pain Relief

**DOI:** 10.3389/fnhum.2019.00279

**Published:** 2019-08-21

**Authors:** Marta Matamala-Gomez, Tony Donegan, Sara Bottiroli, Giorgio Sandrini, Maria V. Sanchez-Vives, Cristina Tassorelli

**Affiliations:** ^1^Neurorehabilitation Unit, IRCCS C. Mondino Foundation, Pavia, Italy; ^2^Department of Brain and Behavioral Sciences, University of Pavia, Pavia, Italy; ^3^Institut d’Investigacions Biomèdiques August Pi i Sunyer (IDIBAPS), Barcelona, Spain; ^4^Faculty of Law, Giustino Fortunato University, Benevento, Italy; ^5^Headache Science Center, IRCCS Mondino Foundation, Pavia, Italy; ^6^ICREA, Barcelona, Spain; ^7^Departament de Cognició, Desenvolupament i Psicologia de l’Educació, Facultat de Psicologia, Universitat de Barcelona, Barcelona, Spain

**Keywords:** embodiment, virtual reality, pain, ownership illusion, body illusion

## Abstract

A significant body of experimental evidence has demonstrated that it is possible to induce the illusion of ownership of a fake limb or even an entire fake body using multisensory correlations. Recently, immersive virtual reality has allowed users to experience the same sensations of ownership over a virtual body inside an immersive virtual environment, which in turn allows virtual reality users to have the feeling of being “embodied” in a virtual body. Using such virtual embodiment to manipulate body perception is starting to be extensively investigated and may have clinical implications for conditions that involve altered body image such as chronic pain. Here, we review experimental and clinical studies that have explored the manipulation of an embodied virtual body in immersive virtual reality for both experimental and clinical pain relief. We discuss the current state of the art, as well as the challenges faced by, and ideas for, future research. Finally, we explore the potentialities of using an embodied virtual body in immersive virtual reality in the field of neurorehabilitation, specifically in the field of pain.

## Introduction

Embodiment is defined as the sense of having a body, and the body can be considered to be both the subject and object of medical science and practice (Gallagher, 2001). One of the main goals in the field of cognitive neuroscience is to investigate how we experience ourselves inside a body as it interacts continuously with the environment. Historically, the bodily self has been described as “obvious and unproblematic” (James, 1890) and connected to a single somatic sensory system such as visceral interception (Damasio, 2000); however, more recently, embodiment has been described as being composed of several different structurally organized subjective components (Longo et al., 2008), as opposed to a single dimension. Hence, we feel our self as being inside a body, a body that moves according to our intentions (Kilteni et al., 2012a) and that interacts with the environment. Indeed, the sense of embodiment is thought to emerge from a complex interaction between bottom–up and top–down signals (Longo et al., 2008).

**FIGURE 1:**
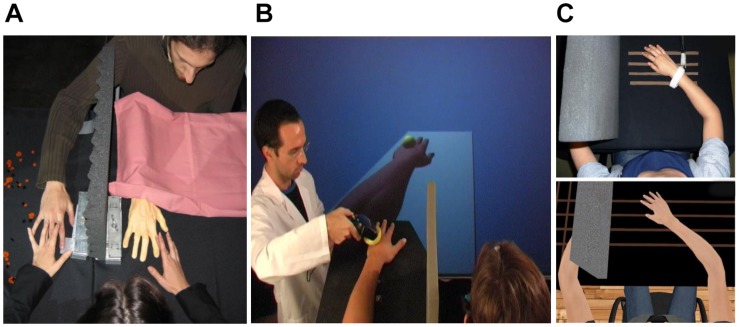
Experimental setups for **(A)** the rubber hand illusion (RHI), **(B)** the virtual hand illusion in non-immersive virtual reality, and **(C)** the virtual hand illusion in immersive virtual reality. Part **(C)** taken from Martini et al. (2015), reprinted with permission from Springer Nature.

At first glance, experimental manipulation of embodiment might seem problematic; however, in the last few years, many studies have investigated bodily perception and revealed alternative ways of manipulating embodiment by using fake body parts. One example of this is the rubber hand illusion (RHI) study, in which synchronous visuotactile stimulation of both a rubber hand located within the visual field of the participant, and the participant’s real hand, located outside the visual field of the participant, confers an illusion of ownership over the rubber hand (Botvinick and Cohen, 1998). Since this study, many researchers have investigated how to manipulate body perception through the use of fake bodies such as a mannequins (Ehrsson and Petkova, 2008), mirrors (Ramachandran et al., 2009a), and virtual reality (VR) (Slater et al., 2008, 2010). Slater et al. (2008) were the first to replicate the RHI study in VR inducing ownership of a virtual hand based on visuo-tactile correlations, in an experience termed the “virtual hand illusion,” while a similar ownership was successfully induced by means of visuomotor correlations in Sanchez-Vives et al. (2010) (see Figure 1). A number of studies have focused on the use of body illusions to address pathological conditions such as chronic pain, with the focus being on the analgesic effects of cross-modal perception (e.g., pain and vision) (for reviews, see Boesch et al., 2015, 2016; Martini, 2016).

Chronic pain, where the symptoms last beyond normal tissue healing times, is the most burdensome health issue worldwide in terms of years lived with disability (Vos et al., 2012) and economic cost (Gaskin and Richard, 2012). In some cases, the negative emotional experience of pain can even lead to suicidal intention (Campbell et al., 2016). Current management strategies including physical activity/exercise and psychological interventions such as cognitive behavioral therapy show short-term effects only, with small effect sizes (Williams et al., 2012; Geneen et al., 2017), while pharmacological agents, such as opioids, have limited efficacy and carry significant risks and side effects (Hofmann et al., 2012; Carter et al., 2014). Indeed, the economic burden of prescription opioid misuse alone in the United States is estimated at $78.5 billion a year, including healthcare costs, lost productivity, addiction treatment, and criminal justice system involvement (Florence et al., 2016). Many investigators have therefore attempted to look for new ways to manage pain states *via* non-pharmacological means (Carter et al., 2014). This paper presents a review of experimental and clinical studies that have explored the manipulation of an embodied virtual body in immersive VR for both experimental and clinical pain relief.

## What is Embodiment?

The capability of our brain of having a representation of our body results in a mental construction composed of perceptions and ideas about the dynamic organization of our own body, involving vision, touch, proprioception, interoception, motor control, and vestibular sensations (Maselli and Slater, 2013). In this regard, embodiment is defined as the sense of having a body. But to what are we referring when we talk about having a body? Longo et al. (2008) described it as follows:

The sense of [having] one’s own body, variously termed “embodiment” (Arzy et al., 2006), “coenaesthesia” (Critchley, 1953), “bodily self-consciousness” (Bermúdez, 1998; Legrand, 2006), or “corporeal awareness” (Critchley, 1979; Berlucchi and Aglioti, 1997), has often been described as a non-conceptual, somatic form of knowledge, different in kind from other types of knowledge (e.g., Kant, 1965; Bermúdez, 1998).

Longo et al. (2008, p. 978)

These different descriptions of embodiment refer to the fact that we are able to feel the sense of having a body by integrating the different sensory signals arriving to our body, which our brain interprets to create a coherent representation of our self. In this regard, Longo et al. (2008) discuss the fact that others have described embodiment as the “storm-center of experience” arriving to our body, resulting in an essential factor for the construction of our internal life (James, 1905), and that other authors support the idea that embodiment is key for the construction of our inner self representation by demonstrating that the sense of embodiment is also closely related to the sense of self, and is strongly related to our individual psychological identity (Edelman, 2005; Cassam, 2012).

However, some investigations have shown that embodiment is divided into different subcomponents that form our body representation, such as body image and body schema (Gallagher and Cole, 1995). In this regard, it is known that body image and body schema play a fundamental, but clearly differentiated, role in understanding the sense of self and in individual psychological identity.

## Conceptual Clarifications of Body Image and Body Schema

Gallagher (2001) has described body image as “an intentional content of consciousness that consists of a system of perceptions, attitudes, and beliefs pertaining from one’s own body.” In contrast, body schema has been described as an “automatic system of processes that constantly regulates posture and movement” and is mostly controlled by the sensorimotor system (Gallagher, 2001). One clear example of the difference between body image and body schema is the difference between perception of movement (conscious awareness of movement), related to body image, and the final execution of that movement (motor performance), related to body schema.

Studies aimed at analyzing body image have distinguished three different intentional elements: (1) the subject’s perceptual experience of his/her own body, (2) the subject’s conceptual understanding of the body, and (3) the subject’s emotional attitude toward his/her own body (Cash and Brown, 1987; Powers et al., 1987; Gardner and Moncrieff, 1988). The body image relies in the congruent inputs for all sensory and motor systems, and it has been described that experimental asynchronous multisensory stimulation results in distortion of body image (Perez-Marcos et al., 2018). In contrast, body schema is not the result of mental perception, beliefs, or attitudes, involving instead a system of motor functions or programs that operate “below” the level of self-referential intentionality, playing a dynamic role in governing posture and movement in a close automatic/subconscious way (Gallagher, 2001). While subconscious and automatic, body schema is not just a matter of mere reflex. Actions controlled by the body schema can be precisely shaped by the intentional experience or goal-directed behavior of one’s own body (Gallagher, 2001). Therefore, once one becomes aware of perceptual limb position, movement, posture, pleasure, pain, and kinesthetic experience, such awareness contributes to the perceptual aspect of one’s body image and such awareness may interact with one’s body schema (Gallagher, 2001).

## The Body in the Brain

According to Melzack (1990), the body schema is controlled by a distributed neural network, or neuromatrix, mostly prewired by genetics, but flexible and open to the continuous shaping influence of experience. This network includes the somatosensory system, reticular afferents to the limbic system, and cortical regions that are important for self-recognition and recognition of external objects and entities. Somatosensory inputs to the brain from different modalities are essential for bodily awareness, especially those from proprioceptors, as demonstrated by Lackner (1988), in which he showed changes in body awareness using muscle vibration and other somatic manipulations. The sense of vision is also very important, as demonstrated by the evident anatomical distortions when congenitally blind subjects attempt to draw their own and other people’s bodies (Critchley, 1979). Further, visual information regarding the hand’s position is normally in accordance with the proprioceptive information regarding its position (van Beers et al., 1999). Tactile events regarding the body are strongly coupled with visual information (if available) of the same event (Pavani et al., 1999). Similarly, execution of movements is normally corroborated by congruent visual and tactile feedback (Janczyk et al., 2009).

### Brain Lesions and Body Representation

In addition to body perception disturbances in congenitally blind subjects, it has also been shown that brain lesions can induce profound changes in body perception and body representation (Aglioti et al., 2016). For example, some patients with right-hemisphere lesions report the delusional perception that their contralateral limb or side of their body does not belong to them—a syndrome called “somatoparaphrenia” (Vallar and Ronchi, 2009; Jenkinson et al., 2013). These types of lesions allow us to explore the relationship between patients’ subjective delusory perceptions and their structural brain deficits (de Vignemont, 2011), especially if those deficits concern areas that are traditionally considered to be multisensory. Further, some brain lesions, such as stroke and/or the resultant neuroplastic changes in the brain, might result in a specific alteration of the body schema or parts of it, as for example in stroke patients who have anosognosia (lack of self-awareness) for their motor and sensory defects and refuse to believe they are affected at all (McGlynn and Schacter, 1989; Levine et al., 1991), or stroke patients with personal neglect (Guariglia and Antonucci, 1992). Disownership of affected body parts can occur after right-sided brain damage (Loetscher et al., 2006), and has also been observed in chronic pain patients suffering from complex regional pain syndrome (CRPS) (Birklein and Schlereth, 2015). In addition, brain-damaged patients without amputations have reported the presence of multiple supernumerary body parts, mostly hands or feet (Halligan et al., 1993; Ramachandran and Blakeslee, 1999). Regarding neuropathic pain patients, limb amputee patients often present with body perception disturbances, such as the affected limb changing in size and form over time (Halligan et al., 1999). Body perception disturbances have also been demonstrated in patients with CRPS (Pleger et al., 2006; Lewis et al., 2007), chronic low back pain (Moseley, 2008), and other chronic pain conditions (Lotze and Moseley, 2007). Finally, body perception disturbances, specifically affecting body image, have been demonstrated in patients with spinal cord injury without brain damage (Fuentes et al., 2013). Part of these body perception disturbances are caused by alterations in the afferent inputs. When a body part is deafferented (deprived of sensory input), the feeling of an increased size of that body part often occurs. Such an effect is observed under local anesthesia, as well as in patients with spinal cord injury that perceived their torso and limbs elongated (Fuentes et al., 2013). Similarly, anomalous multisensory information provided experimentally on the body have been found to elicit a recalibration of the body image with an elongation of the stimulated body part (Perez-Marcos et al., 2018).

In order to study the mechanisms of body perception disturbances, early investigations were conducted in healthy people using devices such as fake limbs, prisms, mirrors, and cameras, which permitted the manipulation of body-related visual cues relative to other body-related sensory information, for example, tactile and proprioceptive cues. On the basis of these techniques, experimental studies on body perception used scenarios in which an external non-self-object was experienced as part of one’s own body through multisensory and/or sensorimotor correlations between the real and the fake body or body part. For many psychologists and neuroscientists, these so-called body ownership illusions (BOIs) have constituted the main experimental method for disentangling body perception in healthy adults over the last 15 years (Blanke et al., 2015).

## Body Ownership Illusions

How the brain represents our body is a fundamental question in cognitive neuroscience (Slater and Sanchez-Vives, 2016). How can we tell that our hand is part of our body and a physical object like a book is not? We generally believe that our own internal body representation is stable; however, some investigations have elicited the illusion of body ownership over objects that are not part of the body at all, which suggests that our body representation is actually highly malleable. In addition, out-of-body illusion research was reignited by Botvinick and Cohen (1998) with their RHI study. In the RHI study, perceived ownership of the rubber hand occurs because the brain’s perceptual system resolves the sensory conflict between the congruent visuotactile information (the visual position of the rubber hand together with the tactile stimulus from the stroking) and the proprioceptive input (which indicates the position of the real hand) by prioritizing the importance of the visuotactile input over the proprioceptive input, integrating the two separate but synchronous inputs (visual and tactile) into a single prediction, as a result of which participants have the perceptual illusion that the rubber hand is their real hand. The visuotactile input is sufficient to override any contradicting proprioceptive input and produce the (incorrect) prediction that the real hand is located closer to where the rubber hand is, a phenomenon known as “proprioceptive drift.” Interestingly, if the visual and tactile stimulation are asynchronous, the illusion does not occur, suggesting that congruous multisensory input is required to produce the illusion. Later, Armel and Ramachandran (2003) demonstrated than when the rubber hand is threatened, there is a strong skin conductance response (SCR), indicating a physiological response to the threat. In this study, they argue that our body representation is continuously updated based on the stimuli being received. With synchronous multisensory perception, we can feel that a rubber hand is our real hand because the brain quickly generates the corresponding illusion as a way of resolving the contradiction between the visuotactile and the proprioceptive inputs (Slater and Sanchez-Vives, 2016).

Further, it has been shown that BOI may also be induced over the entire body in healthy subjects by using a mannequin (Petkova et al., 2011). In this study, healthy subjects observed an artificial body (a mannequin) through a head-mounted display connected to a two-synchronized-color video cameras oriented down at the mannequin body. As in the RHI study and in order to induce a BOI, participants received synchronous visuotactile stimulation at the same place in both the artificial and the real body. This whole body illusion is commonly known as the full BOI (Slater et al., 2010; Maselli and Slater, 2013). The full body ownership illusion from a first-person perspective is described as the feeling of owning an artificial body, which substitutes the real body as the origin of perceptual sensations. In this regard, some investigations have demonstrated that in order to induce a BOI, first-person visual perspective of the artificial body part or full body is key (Ehrsson and Petkova, 2008; Slater et al., 2010; Petkova et al., 2011). In addition to visuotactile stimulation and visual perspective, it has been shown that subjects may also experience the illusion when visuotactile stimulation is substituted by other modalities of multisensory and/or sensorimotor stimulation, such as sensorimotor contingencies in active or passive movements (Tsakiris et al., 2006; Sanchez-Vives et al., 2010; Kalckert and Ehrsson, 2012).

Hence, in the context of full-body illusions, self-location can be advantageously regarded as the combination of two parallel spatial representations: (1) an abstract allocentric representation of the body, mainly associated with visual perspective (first-or third-person visual perspective), and (2) an egocentric mapping of somatosensory sensations (visuotactile or visuomotor sensations) into the external space, mainly associated with peripersonal space. As reported by specific experimental paradigms adopted to induce out-of-body illusions, if these spatial representations are selectively or simultaneously altered, this could have implications for the sense of ownership of an artificial body (Maselli, 2015).

## Embodiment in VR

Nowadays, the integration of technology in the field of applied neuroscience such as VR systems allows the replacement of a person’s real body with a virtual body representation, allowing the subject to feel embodied in a virtual body. In this regard, several investigations demonstrate that one may experience the sense of ownership over a virtual limb (Slater et al., 2008) and even an entire virtual body (Slater et al., 2010) by using immersive VR. In the latter study, Slater and colleagues demonstrated a full-body transfer illusion in which male subjects were able to embody a virtual female body. This finding was demonstrated subjectively (by questionnaire) and physiologically (through heart-rate changes) in response to an attack on the virtual body.

In addition, VR has been defined as a way to simulate reality and real-life situations (Slater and Sanchez-Vives, 2016). For example, it has been demonstrated that when a virtual knife stabs an embodied virtual body in an immersive VR environment, participants demonstrate an autonomic response and motor cortex activation in preparation to move the hand out of the way, just as they would in real life (González-Franco et al., 2014). Hence, anything that can happen in reality can be programed to happen in VR and be experienced as a real situation (Slater and Sanchez-Vives, 2016).

VR allows the experimenter to manipulate not only the virtual environment but also the embodied virtual body in ways that would be impossible in physical reality (Bohil et al., 2011). For example, immersive VR allows the manipulation of body representation in terms of structure, shape, size, and color, in ways that can contrast sharply with our own body image (Kilteni et al., 2012a, b; Banakou et al., 2013; Peck et al., 2013). Further, it has been shown that manipulating the characteristics of the virtual body may influence the physiological responses of the real body (Martini et al., 2013; Bergström et al., 2016), and may also modulate behavioral responses of the subjects (Osimo et al., 2015; Seinfeld et al., 2018). For this reason, immersive VR has been shown to have many potential applications in the fields of psychotherapy, rehabilitation, and behavioral neuroscience (for reviews, see Tarr and Warren, 2002; Martini, 2016; Riva et al., 2018), and even consciousness studies (for a review, see Sanchez-Vives and Slater, 2005).

## VR and Pain Management

At the beginning of the 21st century, VR was introduced to the field of pain management (Hoffman et al., 2000a). The first application of VR in clinical pain was a video game in which adolescent and adult burnt patients experienced less pain while they were playing (Hoffman et al., 2000b). Later, Hoffman and colleagues conducted an fMRI brain scan study in which they found that VR greatly and significantly reduced pain in five brain regions of interest related to pain (the anterior cingulate cortex, primary and secondary somatosensory cortex, insula, and thalamus) in healthy subjects exposed to thermal stimulation (Hoffman et al., 2004). Some years later, a second fMRI study demonstrated that the pain reduction experienced by using VR was comparable to the analgesic effect of a moderate dose of hydromorphone pain medication (Hoffman et al., 2007). Up to this point, the analgesic properties of VR had been mostly attributed to its powerful distractive capacity. However, its effectiveness has been demonstrated in the management of mild and severe pain states (Doctor et al., 2002; Hoffman et al., 2011, 2014). In addition, the positive pain-relieving effects of VR may also be mediated through a reduction in anxiety and through the user experiencing positive emotions such as a sense of fun (Triberti et al., 2014).

One reason in favor of the distractive effect on pain associated to VR in the studies from Hoffman and colleagues is because of the lack of embodiment in a virtual body in the VR scenarios of their studies, in which patients were observing fun and distractive situations in a display instead of being embodied in a virtual environment through an immersive VR system. In addition, Malloy and Milling, in a review on the effectiveness of VR intervention for pain relief, reported that immersive VR is more effective in promoting analgesia than non-immersive VR systems (Malloy and Milling, 2010). The difference between these two systems is the lack of embodiment in the non-immersive VR systems, whereas using immersive VR systems, one may be embodied in a virtual body and immersed in the virtual world, feeling present in the generated VR scenario (Sanchez-Vives and Slater, 2005). It has been reported that this “transportation of consciousness to another place” involved in the sense of presence in a virtual environment might be strong enough to diminish sensations of pain (Sanchez-Vives and Slater, 2005). Hence, although Hoffman and colleagues used an immersive VR system in their pain studies, these early pain studies using VR did not include embodiment in a virtual body.

## Immersive VR and Pain

The sense of being present in an immersive VR scenario while being embodied in a virtual body offers the possibility of modulating pain perception by observing the embodied virtual body from a first-person perspective (for a review, see Martini, 2016). The representation of the body is modulated by the integration of different sensory signals, and this has been extensively investigated (Macaluso and Maravita, 2010; Medina and Coslett, 2010; Serino and Haggard, 2010; Wesslein et al., 2014). In this regard, in IVR, we can therefore act on the virtual body seen from a first-person perspective and experimentally manipulate the multisensory integration in a highly controlled way.

### The Vision of the Body in Pain

It has been shown that watching clips of another person’s hand receiving painful stimuli, while concomitantly receiving painful laser stimulations on one’s one hand, modulates the pain system in the second somatosensory area that reflects the sensory qualities of pain (Valeriani et al., 2008). Later, Longo and colleagues demonstrated, again using laser-evoked potentials, that the vision of one’s painful part of the body is analgesic (Longo et al., 2009). In this study, they conducted three different experiments in which they showed that when participants observed their own painfully stimulated hand (without observing the painful stimulation), they felt less pain compared to when they were looking at a box or at someone else’s hand. The authors postulated that reduction of pain perception while observing one’s own hand was due to a visually induced activation of inhibitory GABAergic interneurons in somatosensory areas. Similarly, Cardini and coworkers showed that vision of the hand, compared to vision of a box, caused a suppression of the early somatosensory potential when electrical stimulation was applied to two fingers at the same time, thus revealing an augmented inhibitory interneuronal activity within the somatosensory cortex (Cardini et al., 2011). This finding was supported by an EEG study by Mancini et al. (2013), in which they demonstrated that vision of the body, compared to vision of a neutral object, increased noxious-related beta oscillatory activity bilaterally in sensorimotor areas, which probably reflects cortical inhibitory activity of nociceptive stimuli processing.

Other neuroimaging studies have found that vision of the painful body part (subjected to painful mechanical stimulations) increases the functional connectivity between brain areas of the so-called “pain matrix” and the posterior parietal and occipito-temporal brain areas related to vision of the body (Longo et al., 2012). Further, in this study, the authors observed that the vision of one’s own hand led to a reduction in the activation of the primary somatosensory cortex and the operculo-insular cortex following painful stimulation (Longo et al., 2012). Specifically, the analgesic effects of the vision of the body part seem to be site-specific, which means that less pain is perceived when looking at the body region where the painful stimuli is applied (Diers et al., 2013). Another factor that modulates pain perception while observing the painful part of the body is visual size modification. One example of this is the study by Mancini et al. (2011), in which the authors found a direct correlation between thermal pain threshold and hand size. Specifically, they found that enlargement of the stimulus-receiving hand enhanced analgesia (i.e., increased the pain threshold), whereas visual reduction of the hand decreased analgesia (reduced the pain threshold). However, there are contradictory results about how visual size modification affects pain perception. For instance, while enlargement of the affected hand had an analgesic effect in healthy subjects (Mancini et al., 2011; Romano et al., 2015), the opposite occurred in patients with chronic arm pain (Moseley et al., 2008), while enlarging the hand had no effect in patients with hand osteoarthritis (Preston and Newport, 2011). In addition, when visual enlargement is shown in a single direction (i.e., a “stretch” illusion) and is accompanied by tactile feedback (emphasizing the stretch by simultaneously pulling on the limb), there is a marked analgesic effect in both hand (Preston and Newport, 2011) and knee osteoarthritis (Stanton et al., 2018). It is worth noting that in both these aforementioned studies, a minority of subjects experienced a greater analgesic effect when the opposite (i.e., a shrink/compression) illusion was shown. The authors suggest that the effect may be specific to the individual (Stanton et al., 2018), which raises the intriguing possibility that greater analgesic effects may be achieved with tailored VR experiences that address cognitive aspects of the patient’s unique pain experience. For example, in osteoarthritis, if patients believe that their pain is caused by compression of the bony surfaces, a stretch illusion may be effective; in other patients who believe that swelling is the primary driver of their pain, a shrink illusion may be more effective.

It has been also shown that the observation of a downscaled back in chronic back pain patients reduced their pain perception, while no effect was reported for the enlarged back visual condition (Diers et al., 2016). The latter study supports the results found in a case study of phantom limb pain conducted by Ramachandran et al. (2009b), in which by using mirrors, they found that minimizing the size of the lost left forearm reduced the patients’ pain perception, while magnifying it had no effect. One explanation for the contradictory results between pain-free participants and chronic pain patients is the complex relationship between pain and the neural representation of the body (Lotze and Moseley, 2007; Gilpin et al., 2015). Related to this, while the temporary painful stimulation in pain-free participants for experimental purposes does not modulate the representation of the body, it is known that patients suffering from chronic pain have associated changes in the central neural system, including a modified cortical representation of the painful part of the body (Moseley and Flor, 2012).

Taken together, these studies demonstrate an important modulatory effect of the vision of one’s own painful part of the body, both in healthy subjects and in subjects with chronic pain. However, it has been recently suggested that, in order to be effective at decreasing pain perception, the visual feedback has to be “realistic” by using real-time video or realistic representations of the painful part of the body, instead of a static or neutral image, at least with chronic lower back pain patients (Diers et al., 2016). For this reason, pain management using immersive VR, which allows subjects to be embodied in a virtual body capable of movement, seems to be a potential alternative for studying pain perception in both healthy and clinical populations.

### Embodiment in VR for Pain Relief

**FIGURE 2:**
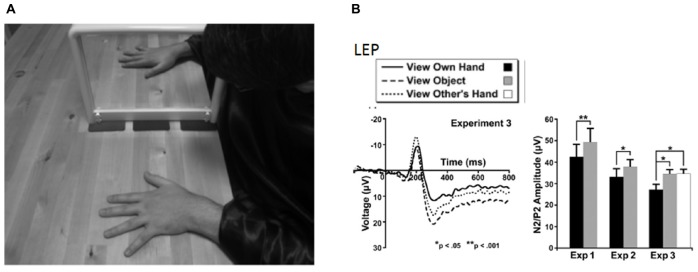
Experimental setup and results from Longo et al. (2009) in which vision of the body was shown to be analgesic, subjectively (using self-report pain ratings) and objectively using laser-evoked potentials. **(A)** The mirror box technique in which the subject has the experience of viewing their right hand, while in fact seeing their left hand reflected in a mirror. **(B)** Laser-evoked potentials (left) and peak-to-peak amplitudes (right) for the three experimental conditions. Error bars are one SEM. Reprinted from Copyright [2009] Society for Neuroscience. ^∗^*p* < 0.05, ^∗∗^*p* < 0.01.

**FIGURE 3:**
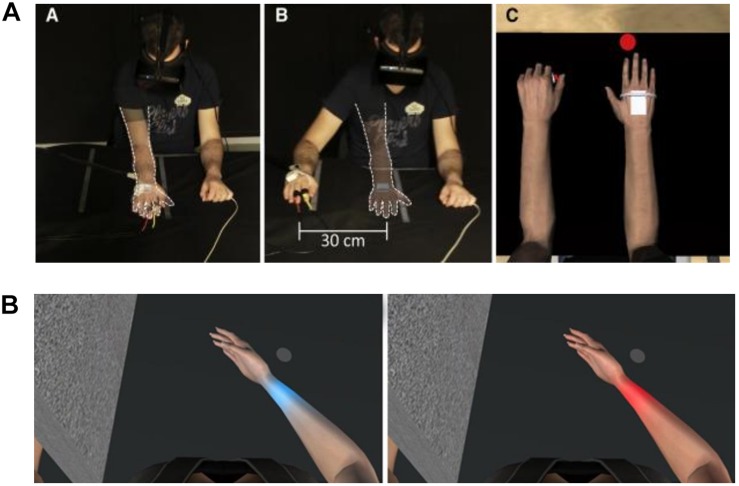
**(A)** Experimental setup of co-location experiment by Nierula et al. (2017). The participant wore a head-mounted display that provided an immersive virtual environment including a virtual own body that was perceived from a first-person perspective. The transparent arm outlined with a white dashed line indicated the positions of the virtual arm. Position of participant during (left panel) co-location, where the virtual and real arm were co-located, and (middle panel) when there was a distance of 30 cm between the real and virtual arm (right panel). The virtual body from the participant’s point of view. Reprinted with permission from Elsevier. **(B)** Participant’s view of virtual arm in the experiment by Martini et al. (2013). The right arm is co-located with the virtual arm, with congruent finger movements, in order to induce embodiment of the virtual limb. Heat stimulation is provided to the wrist while the skin color changed. Pain threshold was increased in the blue arm condition (left) versus the red arm condition (right).

In the context of these studies, Martini and colleagues investigated the effect of virtual body ownership on pain perception and found that looking at one’s own virtual hand also had analgesic properties, as described for the real hand (Longo et al., 2009) (Figure 2). An increased experimental pain threshold was found when compared with the observation of either a real or a virtual object (Martini et al., 2014). Further, they found that the feeling of ownership over the virtual arm was crucial to accomplish the analgesic effect. Regardless, the analgesic effect experienced while observing one’s own body seems to be effective even when observing an embodied virtual body if participants experienced high levels of ownership of the body. The fact that looking at one’s own “rubber hand” (after inducing the RHI) is not analgesic (Mohan et al., 2012) opened up a debate regarding the extent to which looking at a surrogate body was actually analgesic. This issue was sorted out by Nierula et al. (2017), who demonstrated the relevance of the position of the surrogate with respect to the real hand. While the rubber hand cannot be co-located with the real hand (since they both occupy physical space), the virtual hand can be co-located (or not) with the real hand. Nierula et al. (2017) demonstrated that as the distance between the real and the virtual hand increases, the analgesic effect decreases (Figure 3A). In agreement with this, previous findings by Romano and colleagues also reported reduced physiological responses to painful stimuli measured *via* SCRs, when participants observed a virtual body from a first-person perspective co-located with their real body compared with observing the virtual body turned 90° from the real body (Romano et al., 2015). Moreover, in the same study, the authors observed that physiological responses were negatively correlated with the size of the virtual body: the bigger the virtual body, the lower the SCRs (Romano et al., 2015). These results are in line with the observation of a magnified body part increased experimental heat pain thresholds (Mancini et al., 2011; Romano and Maravita, 2014).

Visual manipulations of the body modulate pain perception. One example is the study conducted by Martini et al. (2013) in which the color of a virtual arm was modified and the pain threshold was measured in healthy subjects (see Figure 3B). Specifically, observation of a bluish “cold” virtual arm increased heat pain thresholds, whereas observation of a reddened “hot” virtual arm decreased heat pain thresholds. Co-location of the virtual body with the real one seems to be another key factor for increasing pain thresholds in healthy subjects (Nierula et al., 2017).

**FIGURE 4:**
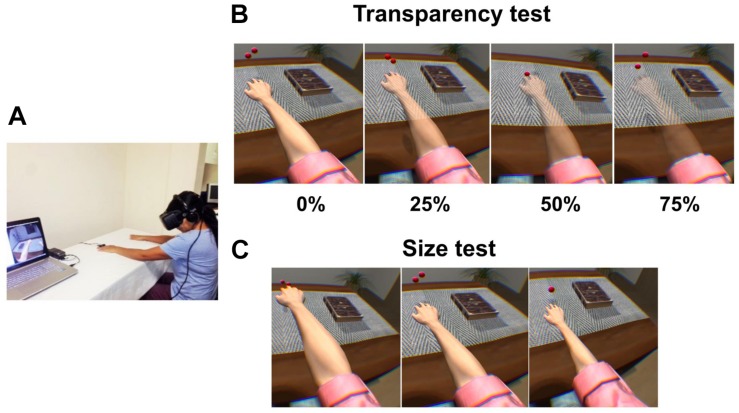
Experimental setup, and transparency and size tests for Matamala-Gomez et al. (2018). **(A)** Patients wore a head-mounted display (HMD) that immersed them in a virtual environment, which allowed participants to feel embodied in a virtual body viewed from a first-person perspective that was co-located with their real body. Virtual balls tapped the fingers during each stimulus presentation, which was accompanied by visuo-tactile stimulation to induce ownership over the virtual arm. **(B)** Transparency test including all four conditions: virtual arm transparency set at 0% (maximum opacity), 25, 50, and 75% (low opacity). **(C)** Size test including all three conditions: virtual arm presented in a big size, in its normal size, and in a small size. Reprinted with permission from Elsevier.

Although evidence suggests that observing one’s own body while experiencing a painful stimulus reduces pain perception, what would happen if the painful part of the body were to fade away? To answer this question, Martini and co-workers conducted an experimental study in which the virtual body was rendered with different levels of transparency while participants were exposed to a painful heat stimulus. They found that the higher levels of transparency were inversely correlated with levels of ownership, but where the body was semi-transparent, higher levels of ownership over a see-through body resulted in an increased pain sensitivity (Martini et al., 2015). Nevertheless, in clinical populations, the effect of transparency is less clear. In this regard, in a study by Matamala-Gomez et al. (2018), two different groups of chronic arm pain patients [CRPS and peripheral nerve injury (PNI)] were immersed in VR and the virtual arm was observed by the patients at four different transparency levels (transparency test) and three different sizes (size test). In contrast to the study conducted on healthy subjects by Martini et al. (2015), Matamala-Gomez et al. (2018) found that increasing transparency levels of the observed virtual arm decreased pain ratings in CRPS, but this did not occur in PNI. Size increase slightly increased pain ratings only in CRPS patients. Further, the authors found that patients with chronic pain can achieve levels of ownership and agency over a virtual arm similar to healthy participants. Moreover, the VR exposure to all of the conditions globally decreased the mean pain ratings by half by the end of the experiment compared to pain ratings at baseline (see Figure 4). This study highlights the possibility that embodiment in VR decreases, at least temporarily, pain ratings in patients with chronic pain. The specific underlying mechanisms of each type of pain probably have a role in the type of strategy that is more effective for reducing pain perception in clinical populations. Further research is required to ascertain optimal dosage and duration of the effects.

Other investigations have also used embodiment in a virtual body to modulate pain perception in clinical populations. In a recent study by Solcà et al. (2018), 24 CRPS patients were immersed in VR, embodied in a virtual body, and observed their affected virtual limb flashing in synchrony with their own detected heartbeat, or asynchronously in the control condition. Here, the authors observed reduced pain ratings and improved motor limb function while observing the synchronous heartbeat condition compared with the asynchronous control condition. Moreover, in another recent study that attempted to modulate neuropathic pain in spinal cord injury patients, the authors showed that VR exposure using multisensory stimulation is associated with mild analgesia, to suggest potential implications for spinal cord injury neurorehabilitation protocols (Pozeg et al., 2017). Finally, Llobera et al. (2013) used body ownership illusions induced using immersive VR combined with a brain–computer interface (BCI) system in a single patient with dystonia of the upper limb suffering from chronic pain. The patient was embodied in a virtual body while observing a virtual hand opening either automatically or through a cognitive task assessed using a BCI that required patient effort. The evaluation was conducted also on a group of five healthy controls. The authors found that embodiment in the virtual body induced changes in electromyography and BCI tasks in the patient that were different from those observed in the controls (see Table 1 for a review).

**TABLE 1:** Summary and characteristics of immersive VR studies using embodiment for pain relief.

**Authors**	**Year**	**Sample**	**Intervention**	**Primary outcomes**
Martini, M., Pérez Marcos, D., and Sanchez-Vives, M. V.	2013	30 healthy participants	The color of the embodied virtual arm was modified (blue, red, or green). Increasing ramps of heat stimulation applied on the participants’ arm were delivered concomitantly with the gradual intensification of different colors on the embodied avatar’s arm.	Reddened arm significantly decreased the pain threshold compared with normal and bluish skin.
Llobera, J., González-Franco, M., Perez-Marcos, D., Valls-Solé, J., Slater, M., and Sanchez-Vives, M. V.	2013	One patient with a fixed posture dystonia of the upper limb. 5 healthy controls.	The virtual hand would open either automatically or through a cognitive task assessed through a BCI that required to focus attention on the virtual hand.	The results reveal that body ownership induced changes on electromyography and BCI performance in the patient that were different from those in five healthy controls.
Martini, M., Perez-Marcos, D., and Sanchez-Vives, M. V.	2014	32 healthy participants	Passive movement of the index finger congruent with the movement of the virtual index finger was used in the “synchronous” condition to induce ownership of the virtual arm. The pain threshold was tested by thermal stimulation under four conditions: (1) synchronous movements of the real and virtual fingers, (2) asynchronous movements, (3) seeing a virtual object instead of an arm, and (4) not seeing any limb in real world.	The ownership of a virtual arm *per se* can significantly increase the thermal pain threshold.
Martini, M., Kilteni, K., Maselli, A., and Sanchez-Vives, M. V.	2015	24 healthy participants	Participants observed four different levels of transparency of the virtual arm (0, 25, 50, and 75%), while they were tested for pain threshold by increasing ramps of heat stimulation.	Body ownership illusion decreases when the body becomes more transparent. Further, providing invisibility of the body does not increase pain threshold.
Romano, D., Llobera, J., and Blanke, O.	2015	21 healthy participants	Participants observed a manipulated visual size (small, normal, big) of an embodied virtual body during painful stimulation.	The results suggest that pain processing is modulated during illusory states of body self-consciousness and that these changes are greater for larger virtual bodies.
Pozeg, P., Palluel, E., Ronchi, R., Solcà, M., Al-Khodairy, A. W., Jordan, X., et al.	2017	20 patients with SCI with paraplegia 20 healthy controls	Participants were submitted to a virtual leg illusion (VLI) and received asynchronous or synchronous visuotactile stimulation to the participant’s back (either immediately above the lesion level or at the shoulder) and to the virtual legs.	Patients with SCI were less sensitive to illusory leg ownership (as compared to HC) and that leg ownership decreased with time since SCI. VLI and full body illusion were both associated with mild analgesia that was only during the VLI specific for synchronous visuotactile stimulation.
Solcà, M., Ronchi, R., Bello-Ruiz, J., Schmidlin, T., Herbelin, B., Luthi, F., et al.	2018	24 patients with CRPS 24 age-and sex-matched healthy controls	Participants were immersed in a virtual environment and shown a virtual depiction of their affected limb that was flashing in synchrony (or in asynchrony in the control condition) with their own online detected heartbeat (heartbeat-enhanced virtual reality).	Heart-enhanced VR reduced pain ratings, improved motor limb function, and modulated a physiologic pain marker (HRV). These significant improvements were reliable and highly selective, absent in control HEVR conditions, not observed in healthy controls.
Matamala-Gomez, M., Gonzalez, A. M. D., Slater, M., and Sanchez-Vives, M. V.	2018	9 patients with CRPS type 1 10 patients with PNI	Participants were immersed in VR and the virtual arm was shown at four different transparency levels (0, 25, 50, 75%), and three sizes (small, normal, big).	All seven conditions globally decreased pain ratings to half. Increasing transparency decrease pain in CRPS but not in PNI. Increasing size increased pain ratings only in CRPS.

## Discussion

This review has discussed the potentialities of using an embodied virtual body in immersive VR for pain modulation. Specifically, we have discussed the use of multisensory integration applications, by means of body ownership illusions, to decrease pain perception in healthy and clinical populations.

In a systematic review conducted by Boesch et al. (2016) of non-virtual body illusions (illusory changes of size, mirror therapy, etc.) on clinical pain, they found that there is limited evidence to suggest that bodily illusions can alter pain, but some illusions, namely, mirror therapy, bodily resizing, and use of functional prostheses, show therapeutic promise. Concerning the effects of embodiment on clinical pain, the authors discuss two studies of patients with chronic pain that showed no effect of embodiment on pain levels and suggest that a potential explanation is that embodiment and pain modulation may be separate processes. However, the review did not examine any studies that used immersive VR studies to induce embodiment. Here, we show that through an embodied virtual body, we may modulate body representation and change pain perception in healthy and clinical populations.

Regarding the importance of body representation in pain perception, it is known that many chronic pain patients have a distorted representation of the affected part of the body (Lewis et al., 2007; Moseley, 2008; Senkowski and Heinz, 2016). Further, misrepresentations of the body have been associated with pain (Lotze and Moseley, 2007), and several reports support structural and functional differences between people with and without pain, both at a cortical or at a subcortical level, in brain areas involved in body awareness and body perception (Flor et al., 1997; Pleger et al., 2006; Gwilym et al., 2010). Distortions of body perception involving a painful part of the body (i.e., the body part feeling larger than it really is) have also been demonstrated (Moyer, 2005; Lewis et al., 2007). There is some evidence that treatment directed at changing these functional brain alterations, such as graded motor imagery and sensorimotor retraining (Moseley, 2004, 2006; Pleger et al., 2006), reduces pain, which suggests that there is a bidirectional link between pain and body perception. In addition to this, it has been shown that pain perception is reduced with a corresponding restoration of functional cortical representation of the painful part of the body in CRPS patients (Pleger et al., 2006).

## Future Research

These studies support a link between body perception and clinical disorders such as pain, highlighting the advantages of using embodiment through VR systems in neurorehabilitation and pain management. Nonetheless, robust and suitably powered randomized control trials are needed to further explore the full potential of body illusions and embodied technologies to modulate pain perception, especially with the use of immersive VR. Furthermore, further investigations aimed at modulating pain perception through an embodied virtual body with larger sample sizes will allow a better understanding of the contribution that the subjective feeling of ownership over an embodied virtual body has on pain perception. Moreover, future studies on this topic may make use of brain imaging techniques, which will allow better identification of the neural structures underlying the complex link between modification of body perception and pain.

Interestingly, virtual body embodiment may also allow the study empathy in pain. It is known that the mere observation of other people in pain tends to elicit empathic responses regarding pain perception in one’s body (Lamm et al., 2011; Benuzzi et al., 2018). Hence, what will happen if we use embodiment to create a pain-free representation of the body? Although some authors have started to investigate how to use empathy for pain relief by using embodiment (Fusaro et al., 2016), further investigations are needed to create new behavioral and cognitive training methods for modulating pain perception in clinical populations.

## Conclusion

The studies commented throughout this narrative review, especially those conducted with chronic pain patients, pave the way for the design of new rehabilitation protocols with prolonged and repeated doses of embodied virtual body in immersive VR to tackle chronic pain disorders, and enable the integration of such “digital therapy” with existing conventional pain treatments.

## Author Contributions

MM-G contributed to the bibliographic review and writing of the manuscript. TD contributed to the writing and review of the manuscript. SB and GS contributed to the bibliographic suggestions and review of the manuscript. MS-V and CT contributed to the supervision of the manuscript.

## Conflict of Interest Statement

The authors declare that the research was conducted in the absence of any commercial or financial relationships that could be construed as a potential conflict of interest.
